# Acquisition Status of Basic Clinical Skills in Japanese Novice Rehabilitation Therapists: A Preliminary Single-Center Study

**DOI:** 10.3390/healthcare11020254

**Published:** 2023-01-13

**Authors:** Kenta Fujimura, Hiroaki Sakurai, Soichiro Koyama, Kazuya Takeda, Takuma Ii, Shota Suzumura, Shigeo Tanabe, Yoshikiyo Kanada

**Affiliations:** Faculty of Rehabilitation, School of Health Sciences, Fujita Health University, Toyoake 470-1192, Japan

**Keywords:** clinical skills, quality, Objective Structured Clinical Examination, occupational therapist, physical therapist, post-graduate training

## Abstract

The number of post-graduate rehabilitation therapists (novice therapists) is increasing due to the growing demand for rehabilitation services in Japan. This study investigated the acquisition status of Japanese novice therapists’ basic clinical skills to clarify their quality and characteristics. Eleven participants’ basic clinical skills (eight physical and three occupational therapists) were assessed using an Objective Structured Clinical Examination. Tasks included exercises of joint range of motion, muscle strengthening, getting up, standing up and sitting down, and transferring between wheelchair and bed. Assessment items were subdivided into categories: attitude, preparation, intervention, safety management, and feedback. One-way ANOVA and Friedman test were used for statistical analysis to compare the data between tasks and categories. The scores for each task’s achievement rate were not statistically significant. However, the achievement rate of each category including tasks was 92.6% (SD 4.0%) for attitude, 81.4% (SD 11.1%) for preparation, 77.9% (SD 14.7%) for intervention, 87.6% (SD 17.3%) for safety management, and 64.0% (SD 14.2%) for feedback. There were significant differences between attitude and feedback (*p* < 0.001), and between safety management and feedback (*p* = 0.012). Post-graduate training programs should focus on improving the quality of clinical skills, especially in skills related to feedback.

## 1. Introduction

The rapid increase in the rate of aging has led Japan to become the world’s first super-aging society. Estimates indicate that the older adult population will account for more than 30% of the national population by 2030 and reach approximately 40% in 2050 [1]. The number of people living with diseases and disabilities has increased alongside the aging rate. The proportion of medical and nursing care expenses under social security benefits is increasing annually [2]. Consequently, there are high social expectations for rehabilitation medicine and its aim to improve mental and physical functioning, the ability to perform activities of daily living (ADL), and quality of life (QOL) for the aged and disabled populations [3].

In 2000, 3048 physical therapists (PT) and 2347 occupational therapists (OT) passed the National Examination for rehabilitation professionals in Japan, while these numbers were 9434 PTs and 4510 OTs in 2021 [4,5,6]. As of 2021, there are 192,327 qualified PTs and 108,814 qualified OTs [4,5,6,7], and due to the recent increase, most related professionals have less than five years of clinical experience, accounting for about 25% of the whole. Advanced clinical skills are necessary to provide optimized and individualized physical or occupational therapy to patients with a wide range of issues related to physical and mental functions, ADL, and QOL. It takes about three years for a new PT to achieve a level of autonomous proficiency, implying that clinical experience is necessary for improving skills [8]. Accordingly, the Ministry of Education, Culture, Sports, Science and Technology has made clinical practice a compulsory part of undergraduate education for rehabilitation therapists [9]. Contents of clinical practice involve observation and practice in clinical settings under the guidance and supervision of practical instructors. However, the clinical skills that clinical practice students learn are limited to “those actions that have been deemed not to be highly mentally or physically invasive” [2,10]. For this reason, more than 90% of the new post-graduate therapists feel that they lack the knowledge and technical skills to start working, although they comply with all requisites for engaging in clinical practice [11]. Therefore, there is a need for post-graduate training on the knowledge, clinical skills, problem solving, and thought processes necessary for clinical practice even after obtaining national certification [12].

To develop an efficient post-graduate training program, it is necessary to identify the new rehabilitation therapists’ clinical skills gained prior to graduation and those that remain underdeveloped. The Objective Structured Clinical Examination (OSCE) [13,14], which is a structured test developed in the 1970s to evaluate clinical skills objectively [15], is widely used in the education of medical professionals [16,17,18,19]; it is also used to evaluate rehabilitation therapists’ basic clinical skills. An OSCE for PTs and OTs (Therapist OSCE) has already been proposed and is now widely used in undergraduate education [20]. The Therapist OSCE text systematically outlines the procedures and key points of basic clinical skills for therapists. It is used to educate and evaluate clinical skills prior to graduation [13]. However, to the best of our knowledge, the only report on the use of the OSCE in evaluating the clinical proficiency of new rehabilitation therapists is Refs. [12,13]. However, the study did not examine the intervention skills of therapists.

This study aimed to evaluate the acquisition status of basic clinical skills to clarify their quality and characteristics among novice therapists. It used the Therapist OSCE for this evaluation to determine the attainment of each skill objectively. The knowledge gained through this research will be key for developing efficient post-graduate training programs for novice therapists in Japan, which may help enhance the quality of rehabilitation care.

## 2. Materials and Methods

This prospective study was approved by the Certified Clinical Research Review Board in our institution (HM21-377) and conducted according to the guidelines of the Declaration of Helsinki.

### 2.1. Participants

The inclusion criterion was post-graduate new physical or occupational therapists who had been working in a general hospital for three months. The exclusion criterion was therapists who did not give consent to participate in this study. The study included eight PTs (four male) and three OTs (three male), all of whom were employed in the rehabilitation ward and had provided written informed consent to participate following a study briefing.

The new therapists performed the following tasks within the first three months of employment. In the first month, they primarily acquired the knowledge and skills necessary for clinical practice through an introduction to the workplace (e.g., learning about employment rules and personal information protection law, handling medical records, etc.), observation, and by providing clinical support to senior therapists. In the second month, they delivered therapeutic practice to patients under the guidance of a therapist with clinical experience. In the third month, physical or occupational therapy plans were developed and delivered to patients autonomously by the new therapists under the supervision of senior, experienced therapists.

### 2.2. Procedure

The Therapist OSCE was conducted based on a textbook [20]. The textbook contains a total of 18 OSCE tasks, among which five basic and frequently used clinical skills were selected: joint range of motion exercise (ROM), muscle strengthening exercise (MS), getting up exercise (Getting up), standing up and sitting down exercise (Standing up), and transferring between wheelchair and bed exercise (Transfer). Test-candidate therapists were afforded five minutes to deliver the exercise to the simulated patient and were scored for clinical skills.

The scoring Items for each task consisted of 15, 15, 12, 18, and 17 items for ROM, MS, Getting up, Standing up, and Transfer, respectively. Scoring items were evaluated on three levels: 0 (Incapable), 1 (Inadequate), and 2 (Capable). Therefore, the total score for each task was 30, 30, 24, 36, and 34, respectively. Two PTs who were familiar with the tasks and scoring of this examination scored the test-candidate therapists; one of them also simulated the role of a patient in the tests. After the examination, the two PTs conferred to determine the participants’ scores.

### 2.3. Analysis

The scoring items for each task were classified into the following five categories: Attitude, Preparation (environment configuration and orientation), Intervention, Safety management, and Feedback. The number of items per category for each task is shown in Table 1. The categories Attitude, Preparation, Intervention, Safety management, and Feedback comprised 15, 14, 37, 5, and 6 items, respectively, with a total score of 30, 28, 74, 10, and 12 points, respectively.

To analyze the results of the Therapist OSCE, the achievement rate for each task, the achievement rate for each category, and the achievement rate for each category of each task were calculated. Statistical analysis was performed using Shapiro–Wilk test to verify normality. Following this, scores were compared using a one-way analysis of variance [21], where normality was confirmed, and a Friedman test [22] was used for non-normality. Regarding significant differences, multiple comparisons were performed; the value of the significant probability was adjusted using a Bonferroni correction [22]. Statistical processing was conducted using SPSS Statistics ver. 27 (IBM, Armonk, NY, USA), and the significance level was 5%.

## 3. Results

### 3.1. Comparison of the Total Scores for Each Task

**Figure 1:**
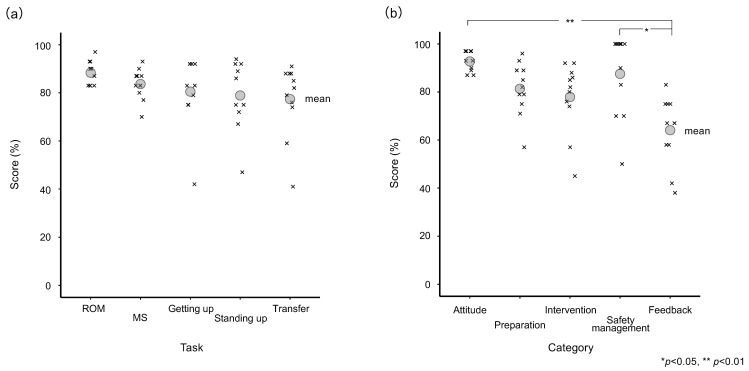
(**a**) Achievement rate for each task; Joint range of movement exercise showed the highest rate, while Transfer showed the lowest rate. (**b**) Achievement rate for each category; Attitude showed the highest rate, while Feedback showed the lowest rate.

The achievement rate for each task is shown in Figure 1a. The achievement rate was 88.5% (standard deviation [SD] 4.8%) for ROM, 83.6% (SD 6.4%) for MS, 80.4% (SD 15.2%) for Getting up, 78.9 (SD 14.7%) for Standing up, and 77.5% (SD 15.2%) for Transfer. There was no statistically significant difference in achievement rates.

### 3.2. Comparison of the Total Scores for Each Category

The achievement rate for each category is shown in Figure 1b. The achievement rate was 92.6% (SD 4.0%) for Attitude, 81.4% (SD 11.1) for Preparation, 77.9% (SD 14.7%) for Intervention, 87.6% (SD 17.3%) for Safety management, and 64.0% (SD 14.2%) for Feedback. A significant difference was found between category scores (df = 4, *p* < 0.001). Multiple comparison results indicated a significant difference between Attitude and Feedback (*p* < 0.001) and between Safety management and Feedback (*p* = 0.012).

### 3.3. Comparison of the Total Score Percentage for Each Category of Each Task

**Figure 2:**
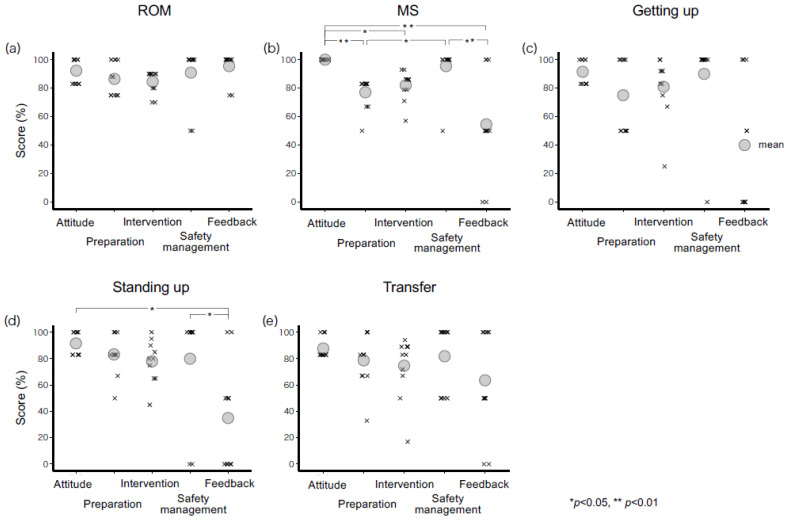
Achievement rate for each classification of each task; The Feedback category showed the lowest rate. Significant differences were indicated between the categories of Muscle strengthening and Standing up and sitting down exercises.

Figure 2 shows the achievement rate for each category of each task. For ROM, there was a significant difference in the achievement rate between categories (df = 4, *p* = 0.035), but there was no significant difference in the multiple comparison. For MS, there was a significant difference in achievement rate between categories (df = 4, *p* < 0.001). Multiple comparison results indicated significant differences between Attitude and Preparation, Intervention, and Feedback (*p* = 0.007, 0.037, 0.002, respectively), Preparation and Safety management (*p* = 0.030), and Safety management and Feedback (*p* = 0.007). For Getting up, there was no significant difference between the categories. Regarding Standing up, there was a significant difference in achievement rate between the categories (df = 4, *p* = 0.002). Multiple comparisons indicated significant differences between Attitude and Feedback (*p* = 0.015) and between Safety management and Feedback (*p* = 0.019). For Transfer, there were no significant differences between the categories.

## 4. Discussion

This research used the Therapist OSCE to determine the rehabilitation therapist’s acquisition status of basic clinical skills soon after becoming employed. Results indicate that the achievement rates for the Getting up, Standing up, and Transfer tasks were not significantly different, albeit lower than those for the ROM and MS, and that individual differences were large. When comparing categories, the achievement rate was the highest for Attitude skills, while Feedback skills had the lowest achievement rate. Similar results were obtained in a previous study that assessed the clinical competence of new therapists using the OSCE [13]; it reported that new therapists tended to have higher attitudinal scores than technical scores.

Developed to assess the basic clinical skills of therapists, the Therapist OSCE provides an objective assessment of basic patient attitude, safety management, and situational feedback skills set in tasks likely to be encountered in clinical practice [15,16,17,18,19]. In our sample, the variation in achievement levels between tasks may have been influenced by differences in the nature and difficulty of the tasks. The ROM and MS tasks exhibited a high average achievement rate trend. In these tasks, the simulated patient was lying in bed with no major postural changes, meaning that test-candidate therapists needed to pay attention mostly to the upper or lower extremities. Meanwhile, the Getting up, Standing up, and Transfer tasks involve a greater postural change from supine to sitting and sitting to standing [20]. Therefore, the test-candidate therapist must be able to facilitate and guide the major postural change of the simulated patient while paying attention to the whole body and focusing on safety management regarding the possibility of falls or tripping. Tasks involving simultaneous movement and cognition are likely to have high difficulty levels since the performance of one or both tasks is likely to decline [23,24].

In the comparative investigation of each category, the achievement rates for Attitude and Safety management were high. For medical and healthcare professionals, basic attitudes and safety management are important topics pertaining to clinical practice in undergraduate education and are set as achievement targets [10,25]. Similarly, in early-stage post-graduate training, the importance of attitudes (e.g., appearance, courteousness, etc.) and safety management to prevent medical accidents are emphasized [12]. Therefore, the achievement rates for Attitude and Safety management were high, possibly due to the great likelihood that the novice therapists continued to receive education and training in related skills. Conversely, achievement rates for Preparation and Intervention skills were low. “Being able to provide treatment under the guidance of a supervisor” is the achievement level goal for therapists who have just gained national qualifications [26]. Therefore, the results suggest that novice therapists and related stakeholders should spend time and energy focusing on Preparation and Intervention skills training.

In clinical practice, there are several instances where rehabilitation therapists have to deal with multiple disabilities of a patient that stem from comorbid disorders. Therefore, post-graduate training may be necessary to optimize the application of basic clinical skills for individual, complex cases. In particular, researchers have suggested that Feedback, which had the lowest achievement rate in our sample, could be an important topic to be emphasized in post-graduate training for rehabilitation therapists. External oral feedback from the therapist and internal feedback (e.g., the individual’s inherent sensations) are important for those undergoing physical or occupational therapy to learn the movements [27]. Given that proper feedback requires timely, language-based communication and immediate interpretation of the patient’s responses and movements, it may be a highly difficult skill for novice therapists to learn.

Qualitative research identified seven categories of abilities that therapists should obtain [28]: “Knowledge required for treatment delivery”, “Clinical thinking skills”, “Medical professional skills for therapists”, “Communication skills”, “Professional attitude”, “Self-learning capability”, and “Self-management capability.” Despite the large number of skills that novice therapists are required to acquire, there are no established standards nor unified programs for measuring their clinical skill levels. Furthermore, the availability of relevant methods for evaluating the clinical skills of novice rehabilitation therapists may be deemed a critical requirement for ensuring the effectiveness of their post-graduate training because such training requires accurate data on their skill levels. As such, various explicit attempts to identify indicators, strategies, and even the clinician growth process have been reported [29,30,31].

This study has several limitations. First, the sample size was small and included both physical and occupational therapists. Moreover, not all participants belonged to the same school, and this led to differences in each school’s training characteristics and the number of hours of clinical practice in the curriculum. Additionally, the research participants were limited to novice therapists in the first three months of employment at a single facility. Therefore, differences in clinical experience among them during the first three months after graduation may have affected the results. Finally, differences in the characteristics of facilities may also have caused the results to differ. Therefore, in the future, the generalizability of these results must be verified with a larger number of participants who graduated from different schools.

## 5. Conclusions

This study clarified the acquisition status of basic clinical skills in novice therapists soon after graduation using the Therapist OSCE. The results suggested that the therapists had high achievement levels for skills emphasized in undergraduate and post-graduate training, such as basic attitudes and safety management. However, their proficiency in skills that require high attentiveness and patient responsiveness during implementation may be insufficient. To improve the quality of clinical skills among novice therapists, post-graduate training programs should be tailored according to their acquisition status of basic clinical skills. Furthermore, since this was a single-center study, further research with a larger number of participants and different institutions is needed in the future.

## Figures and Tables

**Table 1:** Number of items according to each task and category.

Category/Task	ROM ^1^	MS ^2^	Getting Up ^3^	Standing Up ^4^	Transfer ^5^	Total
Attitude	3	3	3	3	3	15
Preparation	4	3	1	3	3	14
Intervention	5	7	6	10	9	37
Safety management	1	1	1	1	1	5
Feedback	2	1	1	1	1	6

^1^ ROM, joint range of motion exercise; ^2^ MS, muscle strengthening exercise; ^3^ Getting up, getting up exercise; ^4^ Standing up, standing up and sitting down exercise; ^5^ Transfer, transfer between wheelchair and bed exercise.

## Data Availability

The data collected and analyzed during the current study are available from the corresponding author on reasonable request.
